# TNF and PGE_2_ in human monocyte-derived macrophages infected with *Chlamydia trachomatis*

**DOI:** 10.1155/S0962935193000511

**Published:** 1993

**Authors:** E. Manor, E. Schmitz, I. Sarov

**Affiliations:** 1 Virology Unit Faculty of Health Sciences Ben-Gurion University of the Negev POB 653 Beer Sheva 84105 Israel; 2 Medizinische Hochschule Hannover Abteilung Rheumatologie Postfach 610180 Hannover 61 D-3000 Germany

## Abstract

In this study levels of prostaglandin E_2_ (PGE_2_), tumour necrosis factor (TNF) and interleukin-1 (IL-1) alpha in medium from monocyte derived macrophages (MdM) infected with *Chlamydia trachomatis* (L_2_/434/Bu or K biovars). TNF and PGE_2_ were found in both cases while IL-1 alpha was not detected. Both TNF and PGE_2_ levels were higher in the medium of the MdM infected with K biovars. TNF reached maximum levels 24 h postinfection, and then declined, while PGE_2_ levels increased continuously during the infection time up to 96 h post-infection. Addition of dexamethasone inhibited production of TNF and PGE_2_. Inhibition of PGE_2_ production by indomethacin resulted in increased production of TNF, while addition of PGE_2_ caused partial inhibition of TNF production from infected MdM.


Mediators of Inflammation 2, 367–371 (1993)

**In this study levels of prostaglandin $E_2$ ($PGE_2$), tumour necrosis factor (TNF) and interleukin-1 (IL-1) alpha in medium from monocyte derived macrophages (MdM) infected with *Chlamydia trachomatis* ($L_2$/434/Bu or K biovars). TNF and $PGE_2$ were found in both cases while IL-1 alpha was not detected. Both TNF and $PGE_2$ levels were higher in the medium of the MdM infected with K biovars. TNF reached maximum levels 24 h post-infection, and then declined, while $PGE_2$ levels increased continuously during the infection time up to 96 h post-infection. Addition of dexamethasone inhibited production of TNF and $PGE_2$. Inhibition of $PGE_2$ production by indomethacin resulted in increased production of TNF, while addition of $PGE_2$ caused partial inhibition of TNF production from infected MdM.**

**Key words:** *Chlamydia trachomatis*, Monocyte derived macrophages, Prostaglandin $E_2$, Tumour necrosis factor

### TNF and $PGE_2$ in human monocyte-derived macrophages infected with *Chlamydia trachomatis*

E. Manor,[1,CA] E. Schmitz[2] and I. Sarov[1]

[1]Virology Unit, Faculty of Health Sciences, Ben-Gurion University of the Negev, POB 653, Beer Sheva 84105, Israel; [2]Medizinische Hochschule Hannover, Abteilung Rheumatologie, Postfach 610180, D-3000 Hannover 61, Germany

[CA] Corresponding Author

### Introduction

Different biovars of *Chlamydia trachomatis*, an obligate intracellular Gram-negative bacterium, have been associated with clinically distinct infections ranging from hyperendemic trachoma (serovars A, B, and C) to sexually transmitted infections and pneumonia (serovars D to K). Lymphogranuloma venereum is a sexually transmitted disease caused by *C. trachomatis* serovars L1, L2 and L3, which are more invasive than biovars D to K. Lymphogranuloma venereum causes a systemic infection characterized primarily by gross lymphadenopathy, suppurative adenitis, and ulcerative genital tract and rectal diseases.[1]

The mononuclear phagocytes, including both the tissue macrophages and their precursors, the circulating blood monocytes, act as effective microbicidal host defence cells against many pathogenic microorganisms. They have been implicated in regulating the functions of lymphoid and haematopoietic cells, and in most cases, these effects are mediated by soluble factors produced by circulating monocytes and tissue macrophages.[2]

Endotoxin and lipopolysaccharide of the outer membrane of Gram-negative bacteria have been found to potently stimulate human monocytes to produce several substances with important biological activities,[3] including interleukin 1 (IL-1),[4,5] tumour necrosis factor (TNF)[6,7] and prostaglandin $E_2$ ($PGE_2$).[8] These factors induce a multitude of biological responses of importance in homeostasis, in host defence mechanisms, and, probably, in the pathogenesis of several diseases.[9–11]

The present study shows that infection of human monocyte derived macrophages (MdM) with $L_2$ or K biovars induces the production of TNF and $PGE_2$ but not of IL-1 alpha. The effect of dexamethasone on the production of these factors in MdM has also been examined.

### Materials and Methods

*Cells:* HEp-2 cells, originating from human carcinoma of the larynx (Flow Laboratories, UK, 03-108) were grown in minimal essential medium (MEM) with glutamine and antibiotics (Biological Industries, Beit Haemek, Israel), and 10% foetal calf serum (FCS) (Gibco Laboratories, Grand Island, NY, USA).

Human monocytes were prepared from heparinized blood of normal donors as described previously.[12] Monocytes grown in RPMI-1640 medium supplemented with 10% heat inactivated foetal calf serum, glutamine and antibiotics, incubated at 37°C in an atmosphere of 5% $CO_2$ for 8–10 days were used as MdM.

*Preparation of purified, infectious EB particles:* Infectious *C. trachomatis* biovar lymphogranuloma venereum (L2/434/Bu) and serum K elementary body particles were purified from BGM cells as described previously.[12]

*Immunoperoxidase assay for titration of* C. trachomatis*: C. trachomatis* was titrated on HEp-2 cells as described previously.[12] The final results of titration were expressed as inclusion-forming units per millilitre.

© 1993 Rapid Communications of Oxford Ltd

*Chlamydial infection in MdM and control cells:* MdM ($8 \times 10^5$ cells/well) and HEp-2 cells ($2 \times 10^5$ cells/well) were grown in 24-well plates (Nunc, Denmark). Twenty-four h later the cells, treated or not treated with either dexamethasone ($10^{-6}$ M), indomethacin ($10^{-6}$ M) or $PGE_2$ ($10^{-6}$ M), were infected or not infected with $L_2$ or K biovars at a multiplicity of infection (M.O.I.) of 1–2. Two h later the unadsorbed *Chlamydiae* were removed, and fresh medium with or without dexamethasone ($10^{-6}$ M), indomethacin ($10^{-6}$ M) or $PGE_2$ ($10^{-6}$ M), respectively, was added to the wells. At various time intervals (2, 24, 48 and 96 h post-infection) medium from the wells was harvested and centrifuged for 5 min at 20 000 × *g*. The supernatant was frozen at −70°C until $PGE_2$, TNF or IL-1 alpha were measured.

*Determination of TNF, $PGE_2$ and IL-1 alpha:* TNF concentrations were determined by ELISA (Biokine TNF Test Kit, T Cell Sciences, Inc., Cambridge, MA, USA). Cell free culture medium from the kinetic studies was subjected to RIA for $PGE_2$ analysis as described previously.[13] IL-1 alpha concentrations were determined by ELISA (Endogen Inc., Boston, MA, USA).

### Results

*$PGE_2$ production from MdM infected with Chlamydiae:* MdM untreated or treated with dexamethasone ($10^{-6}$ M) or indomethacin ($10^{-6}$ M), were infected or mock infected with *C. trachomatis* ($L_2$ or K biovars) at a M.O.I. of 1–2. At various time intervals (2, 24, 48, 96 h post-infection) medium was harvested and $PGE_2$ levels were determined by RIA in the cell-free medium. Fig. 1 shows that the amount of $PGE_2$ in the medium from $L_2$ and K infected MdM increased during the infection time. Infection with the K biovar resulted in higher levels of $PGE_2$ secretion. Infection of the MdM with a higher M.O.I.[5] of both $L_2$ or K biovars resulted in an increase of $PGE_2$ production (data not shown). Daily medium replacement resulted in lower $PGE_2$ production per 24 h from the infected MdM compared with the infected cells in which no medium was replaced (data not shown). Addition of dexamethasone ($10^{-6}$ M) or indomethacin ($10^{-6}$ M) to the chlamydial infected MdM (K or $L_2$) resulted in complete inhibition of $PGE_2$ production. The effect of dexamethasone and indomethacin on $PGE_2$ production observed in MdM infected with the K biovar was much more pronounced than that observed in MdM infected with the $L_2$ biovar. This phenomenon was caused by the reduced ability of the $L_2$ biovar to stimulate $PGE_2$ production in MdM compared to the K biovar. Fig. 2 shows the effect of dexamethasone and indomethacin on $PGE_2$

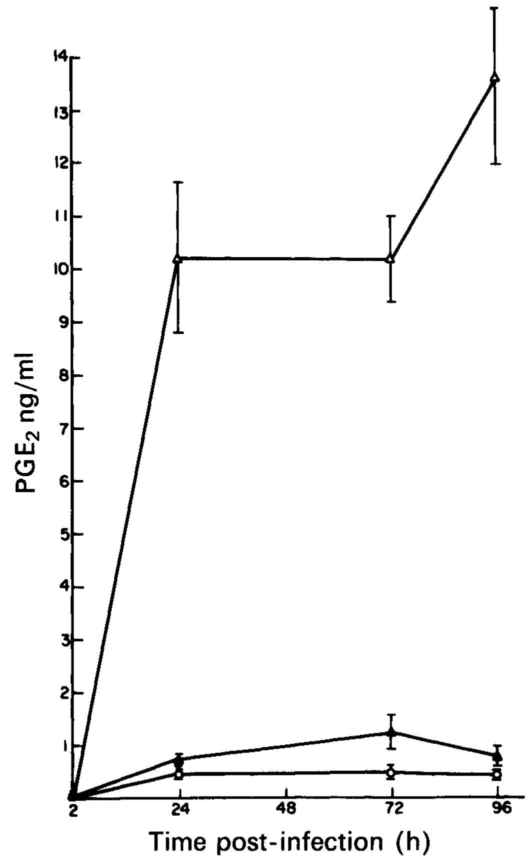

FIG. 1. $PGE_2$ release from MdM infected with *C. trachomatis* $L_2$ (343/Bu) or K biovars at a M.O.I. of 1–2. △, $PGE_2$ release from MdM infected with K biovar; ▲, $PGE_2$ release from MdM infected with $L_2$ biovar; ○, mock infected MdM.

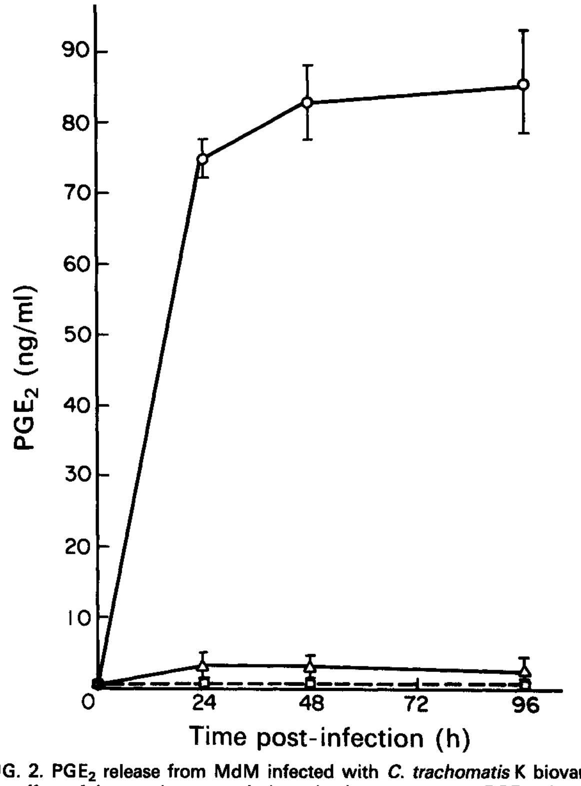

FIG. 2. $PGE_2$ release from MdM infected with *C. trachomatis* K biovar: the effect of dexamethasone or indomethacin treatment. ○, $PGE_2$ release from infected MdM without treatment; △, $PGE_2$ release from infected MdM treated with dexamethasone; □, $PGE_2$ release from infected MdM treated with indomethacin; ---, mock infected MdM.

levels in cell free medium from MdM infected with the K biovar. Medium from control mock infected MdM with or without dexamethasone or indomethacin had low levels of $PGE_2$ (0–0.6 ng/ml).

*TNF production by MdM infected with* Chlamydiae*:* TNF was measured by ELISA in parallel with $PGE_2$ determination. Both $L_2$ and K biovars induced production of TNF by MdM. TNF production was higher in the MdM infected with the K strain. TNF reached maximum levels 24 h post-infection (Fig. 3) and then declined. Daily replacement of the medium from the infected cells with fresh medium resulted in higher levels of TNF (data not shown). Infection at a higher M.O.I.[5] of both $L_2$ and K strains resulted in higher TNF production (data not shown).

Addition of dexamethasone ($10^{-6}$ M) to the *Chlamydia* infected MdM resulted in inhibition of TNF production, while addition of indomethacin ($10^{-6}$ M) resulted in an increase of TNF production. Addition of $PGE_2$ ($10^{-6}$ M) to the *Chlamydia* infected MdM resulted in partial inhibition of TNF production. The effect of dexamethasone and indomethacin on TNF production observed in MdM infected with the K biovar was much more pronounced than that observed in MdM infected with the $L_2$ biovar. Fig. 4 shows the effect of dexamethasone, indomethacin and $PGE_2$ on the TNF level in the medium from MdM infected with biovar K. In medium from mock infected MdM treated or not treated with dexamethasone, indomethacin or $PGE_2$, no TNF was detected.

*Determination of IL-1 alpha in the cell free medium of* chlamydia *infected or mock infected MdM:* The levels of IL-1 alpha in the medium of K or $L_2$ infected or mock infected cells was detected by ELISA in parallel with TNF and $PGE_2$ determination. No difference was found between the IL-1 alpha levels in the mock infected compared to the *Chlamydia* infected MdM medium.

*Chlamydial yield in MdM with or without dexamethasone, indomethacin or* $PGE_2$ *treatment:* MdM were treated or not treated with dexamethasone ($10^{-6}$ M), indomethacin ($10^{-6}$ M) or $PGE_2$ and infected with *C. trachomatis* K and $L_2$ biovars at a M.O.I. of 1–2. Ninety-six h later triplicates of each treatment were harvested and the chlamydial yield was determined. $L_2$ reached a yield of 1–2 × $10^5$ IFU/ml while only 20–50 IFU/ml were detected in the MdM infected with the K biovar. Treatment with indomethacin

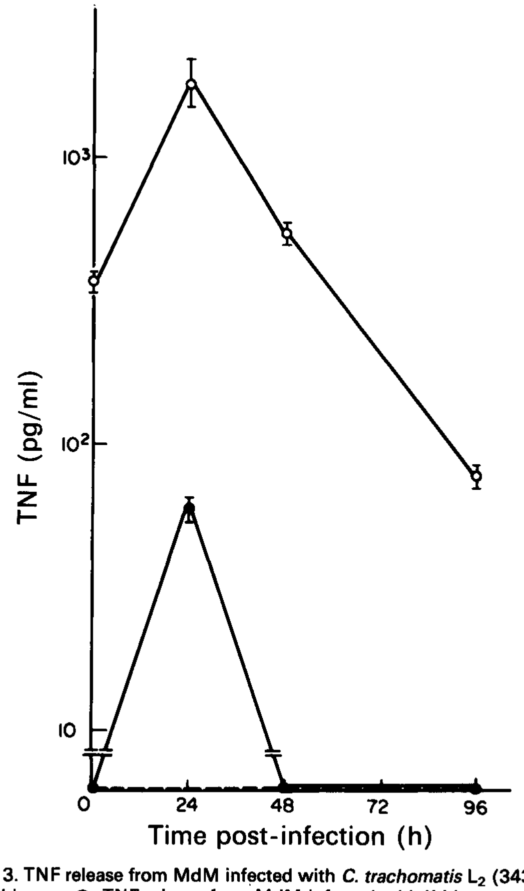

FIG. 3. TNF release from MdM infected with *C. trachomatis* $L_2$ (343/Bu) or K biovars. ○, TNF release from MdM infected with K biovar; ●, TNF release from MdM infected with $L_2$ biovar; ---, TNF release from mock infected MdM.

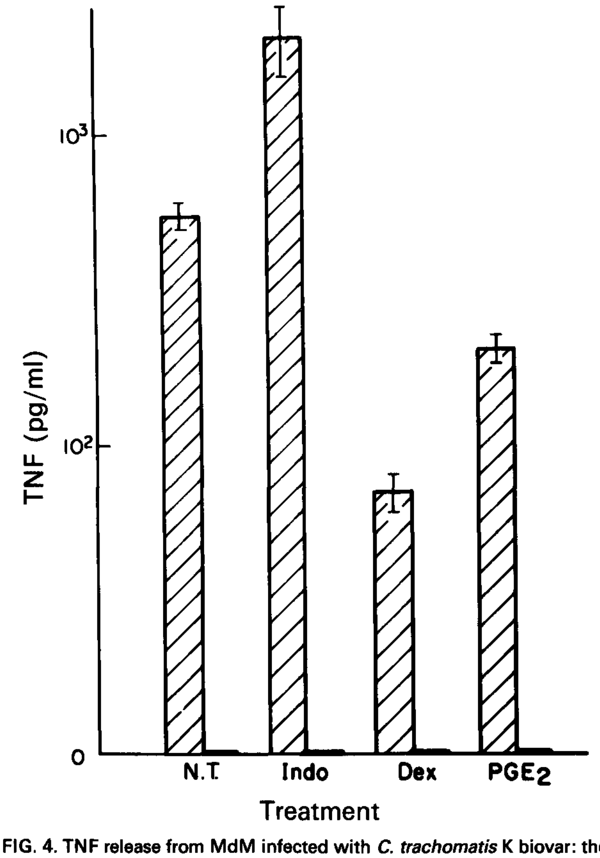

FIG. 4. TNF release from MdM infected with *C. trachomatis* K biovar: the effect of dexamethasone (Dex), indomethacin (Indo) or $PGE_2$, 24 h post-infection. Control without treatment (NT). ▨, Infected cells; ■, control cells.

and $PGE_2$ did not affect chlamydial yield, while treatment with dexamethasone resulted in a 2- to 3-fold increase in chlamydial yield of both K and $L_2$ biovars.

### Discussion

Various biovars of *C. trachomatis* differ in their pathogenicity, but the mechanism is still obscure. One possible explanation might be the ability of the pathogen to turn on host defence mechanisms. The mechanisms of the host defence against diseases caused by chlamydial species are not clearly understood, but both humoral and cell mediated immunity are involved.[14] Infection of macrophages by intracellular parasites might modulate production of TNF, $PGE_2$ and IL-1 alpha, which, in turn, might have a profound effect on the outcome of the infection *in vivo*.

The present study shows clearly that $L_2$ and K biovars induced production of TNF from human MdM (Figs 1 and 3). This production increased when a higher multiplicity of infection of *Chlamydia* was used. Higher levels were observed in the case of K strain infected MdM. This might explain the inability of K strain to grow in MdM, as described by us (Schmitz, Manor, Sarov [Abstract, Germany], and Yong) as well as the difference in the severity of the outcome of the infection *in vivo* between the $L_2$ and K biovars—$L_2$ causing a more generalized infection than K. Recently, Williams *et al.*[15] showed that spleen cells from *C. trachomatis*, pneumonitis agent infected nu/+ and nu/nu mice produced TNF-α. They suggested that TNF-α might play a role in host defence in the murine model. It has been shown that TNF-α inhibits chlamydial growth in HEp-2 cells.[16] This suggests that *in vivo*, early in the chlamydial infection, TNF may play a protective role. However, it is also likely that TNF might cause some of the pathological effects seen in the course of chlamydial infection. The capability of *Chlamydiae* to induce TNF production from macrophages is not unique to chlamydiae, but has been demonstrated recently in a wide range of intracellular pathogens such as viruses,[17] bacteria,[18–20] eukaryotic parasites,[21] and fungi.[22] Various molecules have also been found to be able to induce TNF in macrophages, such as bacterial lipopolysaccharides and endotoxin.[23] Further studies are required to characterize the chlamydial component responsible for the induction of TNF-α in human macrophages.

This study shows that $L_2$ and K also induced human MdM to produce $PGE_2$ (Fig. 1). The level of $PGE_2$ increased when a higher multiplicity of infection of *Chlamydiae* was used. The $PGE_2$ level was higher when MdM were infected with the K strain. $PGE_2$ has been shown to be produced by macrophages infected with viruses,[24,25] bacteria,[26] and intracellular parasites,[27,28] and may cause profound metabolic and functional changes in these cells.[29]

Dexamethasone inhibited TNF and $PGE_2$ production (Figs 2 and 4). These results are in agreement with those described by Beutler *et al.*[30] and by Danon *et al.*,[31] respectively who have shown that dexamethasone inhibits TNF and $PGE_2$ production at the transcriptional level.

Only $L_2$ was found to replicate in MdM.[12] Treatment of the cells with dexamethasone enhanced the yield of infectious chlamydial particles in these cells. These results cannot be simply explained by the dexamethasone inhibition of TNF production from chlamydial infected MdM. This conclusion is based on the findings that addition of an excess of $PGE_2$, which inhibits TNF production, or indomethacin, which enhances TNF production, did not affect the chlamydial yield in MdM. The mechanism by which dexamethasone enhances chlamydial yield in MdM needs further investigation. Corticosteroids have been found to enhance replication of viruses,[32] and certain intracellular parasites.[33–35]

In contrast to $PGE_2$ and TNF-α, no IL-1 was detected in the media from MdM infected with either K or $L_2$ biovars. These results differ from those reported by Rothermel *et al.*,[36] who showed that *C. trachomatis* induced production of IL-1 by human monocytes. This difference might be due to the difference in the M.O.I. used by Rothermel as compared to the M.O.I. in our system (1–2) or to the differentiation state of the cells used by Rothermel (monocytes) compared to ours (MdM). Roux Lombard *et al.*[37] showed that blood monocytes cultured for several weeks, produced much less IL-1 than freshly isolated monocytes. Furthermore, they showed that monocytes cultured for a few weeks produced a specific IL-1 inhibitor.

The level of TNF detected in the medium of the $L_2$ or K strains infected MdM reached a maximum at 24 h post-infection and then declined (Fig. 2). When the infected MdM were washed daily, the TNF level remained high throughout the entire experimental period (data not shown). A possible explanation is that the high level of $PGE_2$ depressed TNF production, and that washing the cells eliminated the interference of $PGE_2$. This explanation is supported by Kunkel *et al.*[8] who showed that $PGE_2$ regulates macrophage derived TNF gene expression. These observations support the suggestion that TNF and $PGE_2$ may affect each other's production.[9,38] TNF produced by activated macrophages may be responsible for increased synthesis of $PGE_2$, which, in turn, limits macrophage activation in an autoregulatory manner.[24,25] A delicate balance between TNF and $PGE_2$ produced by macrophages might play a major role in the outcome and severity of chlamydial infection *in vivo*. Animal model studies are required to examine the possible therapeutic effect of prostaglandin inhibitors and antibodies to TNF on the outcome of chlamydial infections.

### References

1. Ladany S, Sarov I. Recent advances in *Chlamydia trachomatis*. *Eur J Epidemiol* 1985; **1**: 235–256.
2. Nathan CF. Secretory products of macrophages. *J Clin Invest* 1987; **79**: 319–326.
3. Morrison DC, Ulevitch RJ. The effects of bacterial endotoxins on host mediation systems. A Review. *Am J Pathol* 1978; **93**: 526–617.
4. Auron PE, Warner SJC, Webb AC *et al.* Studies on the molecular nature of human interleukin 1. *J Immunol* 1987; **138**: 1447–1456.
5. Dinarello CA. Interleukin-1 and its biologically related cytokines. *Adv Immunol* 1989; **44**: 153–205.
6. Beutler B, Cerami A. Cachectin (tumor necrosis factor): a macrophage hormone governing cellular metabolism and inflammatory response, more than a tumor necrosis factor. *Endocr Rev* 1988; **9**: 57–66.
7. Mannel DN. Biological aspects of tumor necrosis factor. *Immunobiol* 1986; **172**: 2883–2890.
8. Kunkel SL, Spengler M, May MA, Spengler R, Larrick J, Remick D. Prostaglandin $E_2$ regulates macrophage-derived tumor necrosis. *J Biol Chem* 1988; **263**: 5380–5384.
9. Lehmmann V, Benninghoff B, Droge W. Tumor necrosis factor-induced activation of peritoneal macrophages is regulated by prostaglandin $E_2$ and cAMP. *J Immunol* 1988; **141**: 587–591.
10. Bernheim HA. Is prostaglandin $E_2$ involved in the pathogenesis of fever? Effects of interleukin-1 on the release of prostaglandins. *Yale J Biol Med* 1986; **59**: 151–158.
11. Nichols FC, Garrison SW, Davis HW. Prostaglandin $E_2$ and thromboxane $B_2$ release from human monocytes treated with bacterial lipopolysaccharide. *J Leukoc Biol* 1988; **44**: 376–384.
12. Manor E, Sarov I. Fate of *Chlamydia trachomatis* in human monocytes and monocyte-derived macrophages. *Infect Immun* 1986; **54**: 90–95.
13. Danon A, Leibson V, Assouline G. Effects of aspirin, indomethacin, flufenamic acid and paracetamol on prostaglandin output from rat stomach and renal papilla *in vitro* and *ex vivo*. *J Pharm Pharmacol* 1983; **35**: 576–579.
14. Lamont HC, Nichols RL. Immunology of chlamydial infection. In: Nahmias AJ, O'Reilly J, eds. *Immunology of Human Infection*. New York: Plenum Publishing Corp., 1981; 441–474.
15. Williams DM, Bonewald LF, Roodman GD, Byrne GI, Magee DM, Schachter J. Tumor necrosis factor alpha is a cytotoxin induced by murine *Chlamydia trachomatis* infection. *Infect Immun* 1989; **57**: 1351–1355.
16. Shemer-Avni Y, Wallach D, Sarov I. Inhibition of *Chlamydia trachomatis* growth by recombinant tumor necrosis factor. *Infect Immun* 1988; **56**: 2503–2506.
17. Aderka D, Holtmann H, Toker L, Hahn T, Wallach D. Tumor necrosis factor induction by Sendai virus. *J Immunol* 1985; **136**: 2938–2942.
18. Havel EA. Production of tumor necrosis factor during murine listeriosis. *J Immunol* 1987; **139**: 4225–4231.
19. Izumi S, Hirai O, Kayashi K, Kohsaka HA, Yamamura Y. Induction of a tumor necrosis factor-like activity by *Nocardia rubra* cell wall skeleton. *Cancer Res* 1987; **47**: 1785–1792.
20. Leist TP, Frei K, Kam-Hansen S, Zinkernagel RM, Fontana A. Tumor necrosis factor alpha in cerebrospinal fluid during bacterial, but not viral, meningitis. *J Exp Med* 1988; **167**: 1743–1748.
21. Bate CAW, Taverne J, Playfair JHL. Malarial parasites induce TNF production by macrophages. *Infect Immunol* 1988; **64**: 227–231.
22. Djeu JY, Blanchard DK, Richards AL, Friedman H. Tumor necrosis factor induction by *Candida albicans* from human natural killer cells and monocytes. *J Immunol* 1988; **141**: 4047–4052.
23. Jupin C, Anderson S, Damais C, Alouf JE, Parant M. Toxic shock syndrome toxin 1 as an inducer of human tumor necrosis factors and gamma interferon. *J Exp Med* 1988; **167**: 752–761.
24. Laegreid WW, Taylor SM, Leid RW *et al.* Virus-induced enhancement of arachidonate metabolism by bovine alveolar macrophages *in vitro*. *J Leukoc Biol* 1989; **45**: 283–292.
25. Laegreid WW, Liggitt HD, Silflow RM, Evermann JR, Taylor SM, Leid RW. Reversal of virus-induced alveolar macrophage bactericidal dysfunction by cycloxygenase inhibition *in vitro*. *J Leukoc Biol* 1989; **45**: 293–300.
26. Molvig J, Baek L, Christensen P *et al.* Endotoxin-stimulated human monocyte secretion of interleukin 1, tumour necrosis factor alpha, and prostaglandin $E_2$ shows stable interindividual differences. *Scand J Immunol* 1988; **17**: 705–714.
27. Clark IA, Hunt NH. Increased production of arachidonate metabolites by peritoneal cells of mice infected with *Plasmodium vinckei vinckei*. *Aust J Exp Biol Med Sci* 1986; **64**: 415–418.
28. Reiner NE, Schultz LA, Malemud CJ. Eicosanoid metabolism by *Leishmania donovani*-infected macrophages: mouse strain responses in prostanoid synthesis. *Am J Trop Med Hyg* 1988; **38**: 59–64.
29. Chouaib S, Beroglio JH. Prostaglandins E as modulators of the immune response. *Lymphokine Res* 1988; **7**: 237–245.
30. Beutler B, Krochin N, Milsark IW, Luedke C, Cerami A. Control of cachetin (tumor necrosis factor) synthesis: mechanisms of endotoxin resistance. *Science* 1986; **232**: 977–978.
31. Danon A, Assouline G. Inhibition of prostaglandin biosynthesis by corticosteroids requires RNA and protein synthesis. *Nature* 1978; **273**: 552–554.
32. Tanaka J, Ogura T, Kayiya S *et al.* Enhanced replication of human cytomegalovirus in human fibroblasts treated with dexamethasone. *J Gen Virol* 1984; **65**: 1759–1767.
33. Bushell AC, Hobson D. Effect of cortisol on the growth of *Chlamydia trachomatis* in McCoy cells. *Infect Immun* 1978; **21**: 946–953.
34. Woodman DR, Schultz WW, Woodman KL, Weiss E. Growth of *Rickettsia typhi* in irradiated L cells enhanced by lysosomal stabilization. *Infect Immun* 1979; **23**: 61–67.
35. Yang YS, Kuo CC, Chen WJ. Reactivation of chlamydia trachomatis lung infection in mice by cortisone. *Infect Immun* 1983; **39**: 655–658.
36. Rothermel CD, Schachter J, Lavrich P, Lipsitz EC, Francus T. *Chlamydia trachomatis*-induced production of interleukin-1 by human monocytes. *Infect Immun* 1989; **57**: 2705–2711.
37. Roux-Lombard P, Modoux C, Cruchaud A, Dayer JM. Purified blood monocytes from HIV-infected patients produce high levels of TNF alpha and IL-1. *Clin Immunol Immunopathol* 1989; **501**: 374–384.
38. Dayer JM, Beutler B, Cerami A. Cachectin/tumor necrosis factor stimulates collagenase and prostaglandin E2 production by human synovial cells and dermal fibroblasts. *J Exp Med* 1985; **162**: 2163–2168.

ACKNOWLEDGEMENT. We thank Maureen Friedman for critical review of the manuscript.

**Received 30 June 1993; accepted 22 July 1993**
